# Cancer incidence in Khartoum, Sudan: first results from the Cancer Registry, 2009–2010

**DOI:** 10.1002/cam4.254

**Published:** 2014-05-13

**Authors:** Intisar E Saeed, Hsin-Yi Weng, Kamal H Mohamed, Sulma I Mohammed

**Affiliations:** 1 Cancer Registry Center, Federal Ministry of HealthKhartoum, Sudan; 2 Department of Comparative Pathobiology, Purdue UniversityWest Lafayette, Indiana; 3 Radiation and Isotopes Center KhartoumAlgaser Street, Khartoum, Sudan; 4 Purdue University Center for Cancer Research and Department of Comparative Pathobiology, Purdue UniversityWest Lafayette, Indiana

**Keywords:** Cancer, incidence, Khartoum, sub-Saharan Africa, Sudan

## Abstract

In 2009, the first National Population-based Cancer Registry (NCR) was established in Sudan. We report in this study, the first data from the NCR for Khartoum State for the period 2009–2010. The NCR staff used passive and active approaches to collect data on cancer diagnosed by all means in Khartoum State. Rates were age standardized to the 2010 Sudan Standard Population and 1966 and 2000 World Standard Population and expressed per 100,000 populations. During 2009–2010, 6771 new cancer cases were registered. Of those, 3646 (53.8%) cases were in women and 3125 (46.2%) were in men. The most commonly diagnosed cancer among women was breast followed by leukemia, cervix, and ovary, and among men it was prostate cancer followed by leukemia, lymphoma, oral, colorectal, and liver. In children less than 15 years of age, leukemia was the most common cancer followed lymphoma, and cancer of the eye, bone, kidney, and the brain. The overall age-standardized rate (ASR) per 100,000 population was higher in women (124.3) than in men (90.8) using 2010 Sudan Standard Population. Similarly, it was higher in women (188.6 and 206.3 per 100,000 population) than in men (145.4 and 160.0 per 100,000 population) using 1966 and 2000 World Standard Population, respectively. The data from NCR indicated that prostate and breast as the most commonly diagnosed cancer sites in men and women in Khartoum, while cancer of the cervix trailed behind portraying a cancer picture similar to that of the developed world. Despite the study limitations, the NCR data gave a fair representation of cancer profile of Khartoum State and underscored the need for high-quality cancer registries in Sudan.

## Introduction

Cancer is becoming a global health problem and the number of cancer cases in sub-Saharan Africa is rising. Being an African country, Sudan has its share of cancer burden. However, population-based data in cancer incidence, prevalence, and mortality in Sudan were not available and most published cancer cases were based on estimates from hospital-based information sources. Most of these sources are maintained by individual health institutions and are mostly paper based. The National Health Laboratories is the main diagnostic laboratory in Khartoum and its cancer database captures histopathologically confirmed cases 1. Another cancer data source is the Radiation and Isotope Center at Khartoum (RICK), Khartoum State, which treated as well as diagnosed the disease using radioactive isotope. Recently the center expanded to include nuclear as well as clinical departments with all necessary cancer expertise. The center receives referrals from all over the country. The third national data source is the National Cancer Institute, University of Gezira, which is located at Wadmadani capital of Gezira State. It provides medical care for cancer patients from Gezira State as well as the surrounding states in the central region of Sudan.

Recently, in 2009, Sudan established the first National Cancer Registry (NCR). NCR is to develop a system that will facilitate creation and maintenance of local and regional data and assemble these data into a single centrally accessible system. In the hope, that the NCR will provide data on burden of cancer in Sudan allowing policy makers to implement cancer control measures and prevention strategies. In this study, we present the results from the Cancer Registry for Khartoum State for the period 2009–2010.

## Material and Methods

**Figure 1:**
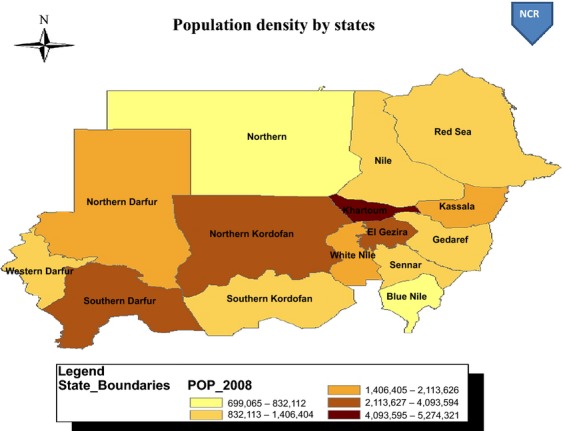
Sudan population distribution as of 2008.

The Sudan is consisted of 15 states with varying population densities (Fig.1). The public sector health services in Sudan are organized at three levels primary, secondary, and tertiary. The states' general hospitals are the referral centers for the entire state. Specialized centers and Khartoum General Hospital, located in capital Khartoum, constitute the tertiary level. After exhausting all the medical attempts for treatment at the primary and secondary care facilities as well as local healers, patients are referred to RICK. The NCR is established under the auspices of Ministry of Federal Health and is located in the capital Khartoum. The registry building is located in the medical complex neighboring the Khartoum Teaching Hospital, RICK, and other clinics and laboratories that provide cancer management and diagnosis services.

### Cancer data

The Sudan NCR abstracts and collects cancer data from all public and private health institutions that provide cancer laboratory diagnosis and treatment, as well as the data from RICK. The registry uses two methods for data collection, active and passive case finding, covering about 79 private and governmental heath facilities and pathological laboratories (18 histopathological laboratories, 33 private, and 28 public hospitals). Sources of data include laboratory reports (histopathology, hematology, and biochemistry), medical record ledgers, theater books, patient case notes, and ward admission. Following variables are collected for each case: name, age, gender, nationality, tribe, residence, state of residence, date of diagnosis, tumor site, tumor histology, basis of diagnosis, occupation, medical record number, and place of referral. The data used for this study were obtained from the patients residing in Khartoum State during the 2-year period 2009–2010. Care was taken to distinguish residents of Khartoum from out-of-state patients that used relatives' addresses.

### Population data

**Figure 2:**
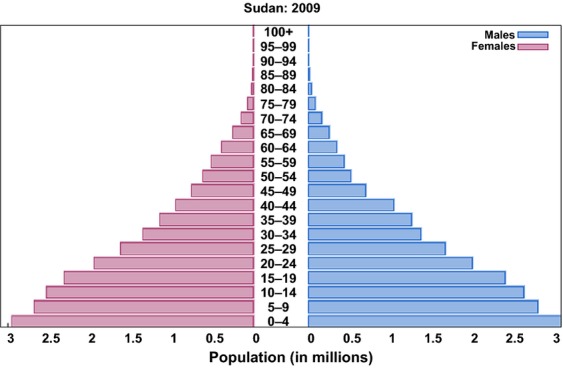
Sudan population pyramid 2009.

Khartoum State has an area of 22,122 km^2^ and an estimated urban/rural population of approximately 7 million according to 2008 census and it has structure similar to that of the entire country (http://www.cbs.gov.sd). The 2009 projected Khartoum state populations (by gender and age groups [0–14 years old, 15–24 years old, 25–54 years old, 55–64 years old, and 65 years and older]) was used for computing the incidence rates (Fig.2).

### Quality control

NCR team applies different methods of data checking, communication, field visits, and reviewing prior entering the data electronically, in order to complete missing data on cancer collected from different health sectors and to avoid multiple registrations and duplication. After the initial checking, the data were then entered into the computer using CanReg-5 software developed by IARC, Lyon, France. The software has a duplicate entry checking capability and validity checks. Tumor site and morphology are coded according to the 10th edition of International Classification of Diseases (ICD) for Oncology 2. The data were then exported to IBM® SPSS® Statistics (version 20.0; IBM Corp., Armonk, NY) for further analyses. The data were checked again for out-of-range values, typos, and incompatible records by two independent investigators.

### Statistical analysis

Crude as well as gender- and age-specific incidence rates for each cancer site were derived. Age was categorized into five groups as previously described for age-specific rates. Direct method was used to compute age-standardized rates (ASR) using different standard populations, including the 2010 Sudan population (http://www.cbs.gov.sd) and the 1966 3 and 2000 World Standard Population (WSP) 4.

## Results

Total of 6771 incident cases of cancer were recorded among Khartoum residents in 2009–2010. Among them, 3125 (46.2%) were men and 3646 (53.8%) were women. Of those who had information on age at diagnosis (*N* = 6711), 486 (7.2%) were children aged less than 15 years, 319 (4.8%) were between 15 and 24 years, 2849 (42.5%) were between 25 and 54, 1227 (18.3%) were between 55 and 64, and 1830 (27.3%) were 65 years and older.

The majority (59.83%) of the cases were microscopically verified (MV). Registrations were considered MV where diagnosis was based on malignant histological or cytological reports. About 4.03% of cases were diagnosed by clinical investigations (X-ray, endoscopy, imaging, ultrasound, surgery, and autopsy), 0.33 diagnosed clinically, 24.6% were diagnosed by laboratory examinations, and 0.04% were diagnosed by death certificate (DCO%) and for about 12.1% diagnostic methods were not recorded. The cancer sites of 223 cases (3.4%) were unknown.

### Top 10 most common primary cancer sites in Khartoum

**Figure 3:**
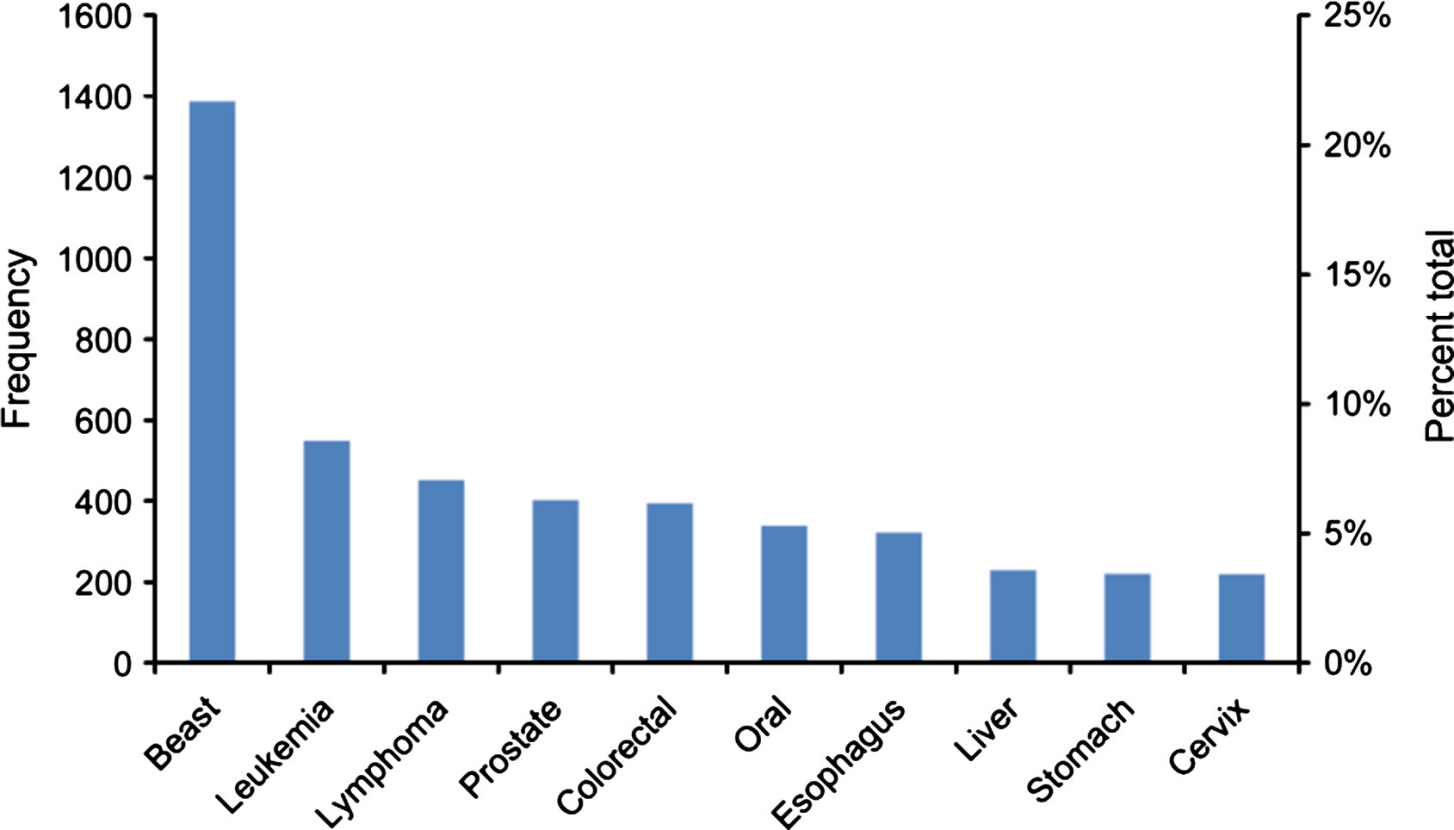
Top 10 most common primary sites in Khartoum, Sudan: 2009–2010. *N *=* *6548.

The top 10 most common cancer sites in Khartoum are shown in Figure3. Among all registered cancer cases with available information (*N* = 6548, 96.7%), breast cancer was the most common cancer with an incidence rate of 25.1 per 100,000, followed by leukemia (rate = 10.0 per 100,000), lymphoma (rate = 8.2 per 100,000), prostate cancer (rate = 7.3 per 100,000), colorectal cancer (rate = 7.1 per 100,000), oral cancer (rate = 6.1 per 100,000), cancer of esophagus (rate = 5.8 per 100,000), liver cancer (rate = 4.2 per 100,000), stomach cancer (rate = 4.0 per 100,000), and cancer of cervix (rate = 4.0 per 100,000). These cancers together made up 68.9% of all reported primary cancer sites during 2009–2010.

### Gender- and age-specific cancer incidence rates

**Figure 4:**
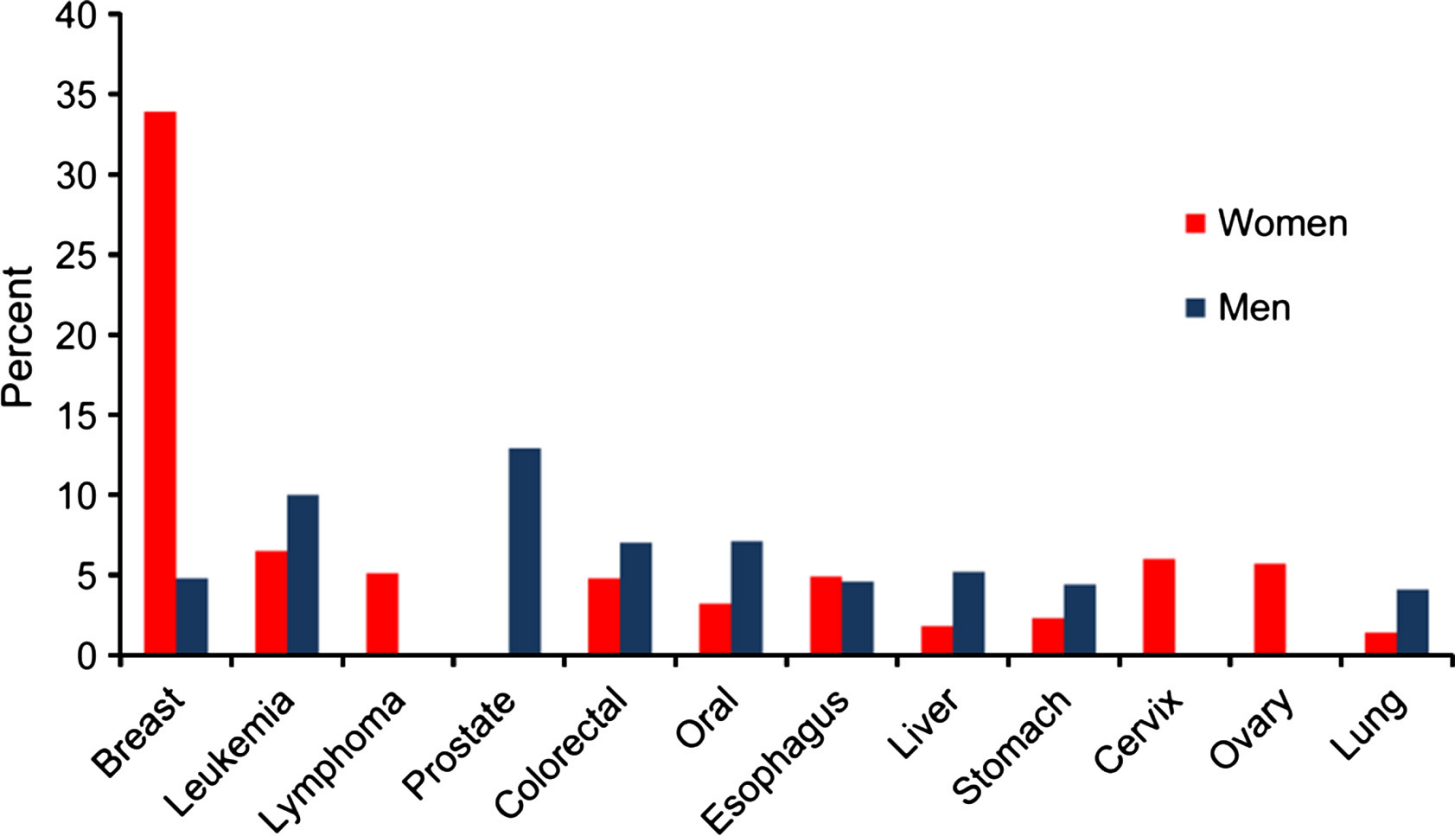
Most common primary cancer sites by gender in Khartoum, Sudan. *N* = 6771.

Figure4 shows the most common cancer sites by gender in Khartoum during the study period. In women, of the 3646 cases (incidence rate = 140.8 per 100,000) registered for 2009–2010, breast cancer was the most common cancer (gender-specific rate = 47.8 per 100,000), followed by leukemia (gender-specific rate = 9.1 per 100,000), cancer of the cervix (gender-specific rate = 8.5 per 100,000), cancer of the ovary (gender-specific rate = 8.0 per 100,000), lymphoma (gender-specific rate = 7.2 per 100,000), cancer of esophagus (gender-specific rate = 6.8 per 100,000), and colorectal cancer (gender-specific rate = 6.8 per 100,000). In men, of 3125 cases (incidence rate = 106.8 per 100,000) registered during the 2009–2010 period, prostate cancer was the most common cancer (gender-specific rate = 13.7 per 100,000), followed by leukemia (gender-specific rate = 10.7 per 100,000), lymphoma (gender-specific rate = 9.1 per 100,000), oral cancer (gender-specific rate = 7.6 per 100,000), colorectal cancer (gender-specific rate = 7.5 per 100,000), and cancer of the liver (gender-specific rate = 5.5 per 100,000).

There are differences in the distribution of the most common cancer sites in children less than 15 years of age (Table1) and in adults 15 years and older (Table2). Leukemia (age-specific rate = 9.3 per 100,000) was the most common cancer in children (Table1) while breast cancer (age-specific rate = 37.8 per 100,000) was the most common cancer in adults (Table2). In adults 15 years and older, for all cancer sites, the cancer incidence rate increased with age.

**Table 1:** The top 10 most common cancer sites in children younger than 15 years, Khartoum, Sudan: 2009–2010.

Cancer site	No.	Rate
Leukemia	179	9.3
Lymphoma	65	3.4
Eye	42	2.2
Bone	34	1.8
Kidney	23	1.2
Brain	18	0.9
Breast	18	0.9
Oral	15	0.8
Liver	8	0.4
Stomach	7	0.4
Total	486	25.4

Rate: per 100,000.

**Table 2:** The top 10 most common cancer sites in adults aged 15 years or older, Khartoum, Sudan: 2009–2010.

Cancer site	15–24		25–54		55–64		65+		Total	
	No.	Rate	No.	Rate.	No.	Rate	No.	Rate	No.	Rate
Breast	37	3.2	884	44.0	215	92.4	223	113.2	1359	37.8
Colorectal	17	1.5	198	9.9	64	27.5	110	55.8	389	10.8
Prostate	2	0.2	57	2.8	71	30.5	257	130.5	387	10.8
Lymphoma	49	4.2	148	7.4	90	38.7	97	49.2	384	10.7
Leukemia	35	3.0	176	8.8	67	28.8	90	45.7	368	10.2
Oral	11	0.9	138	6.9	76	32.7	98	49.8	323	9.0
Esophagus	7	0.6	119	5.9	73	31.4	121	61.4	320	8.9
Liver	3	0.3	80	4.0	55	23.6	81	41.1	219	6.1
Cervix	4	0.3	100	5.0	53	22.8	55	27.9	212	5.9
Stomach	8	0.7	76	3.8	53	22.8	71	36.0	208	5.8
Total	319	27.5	2849	142.0	1,226	526.8	1830	929.0	6224	173.0

Rate: per 100,000.

**Figure 5:**
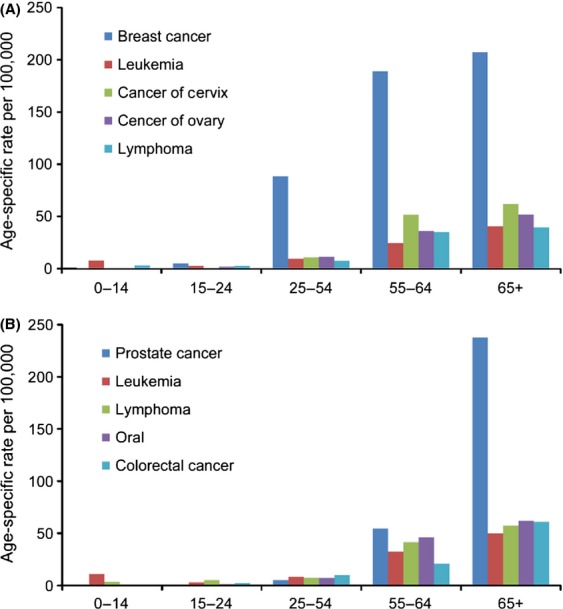
(A) Top most common primary cancer sites in women by age group, Khartoum, Sudan: 2009–2010. *N* = 3619. (B) Top five most common primary cancer sites in men by age group, Khartoum, Sudan: 2009–2010. *N* = 3619.

When stratified by both gender and age, the incidence rate of breast cancer was substantially higher than the other cancer sites in women aged 25 years and older (Fig.5A) while prostate cancer dominated among men aged 65 years and older (Fig.5B). A positive association between incidence rate of the top five most common cancer sites and age was observed in both adult women and men.

### Age-standardized rates

**Figure 6:**
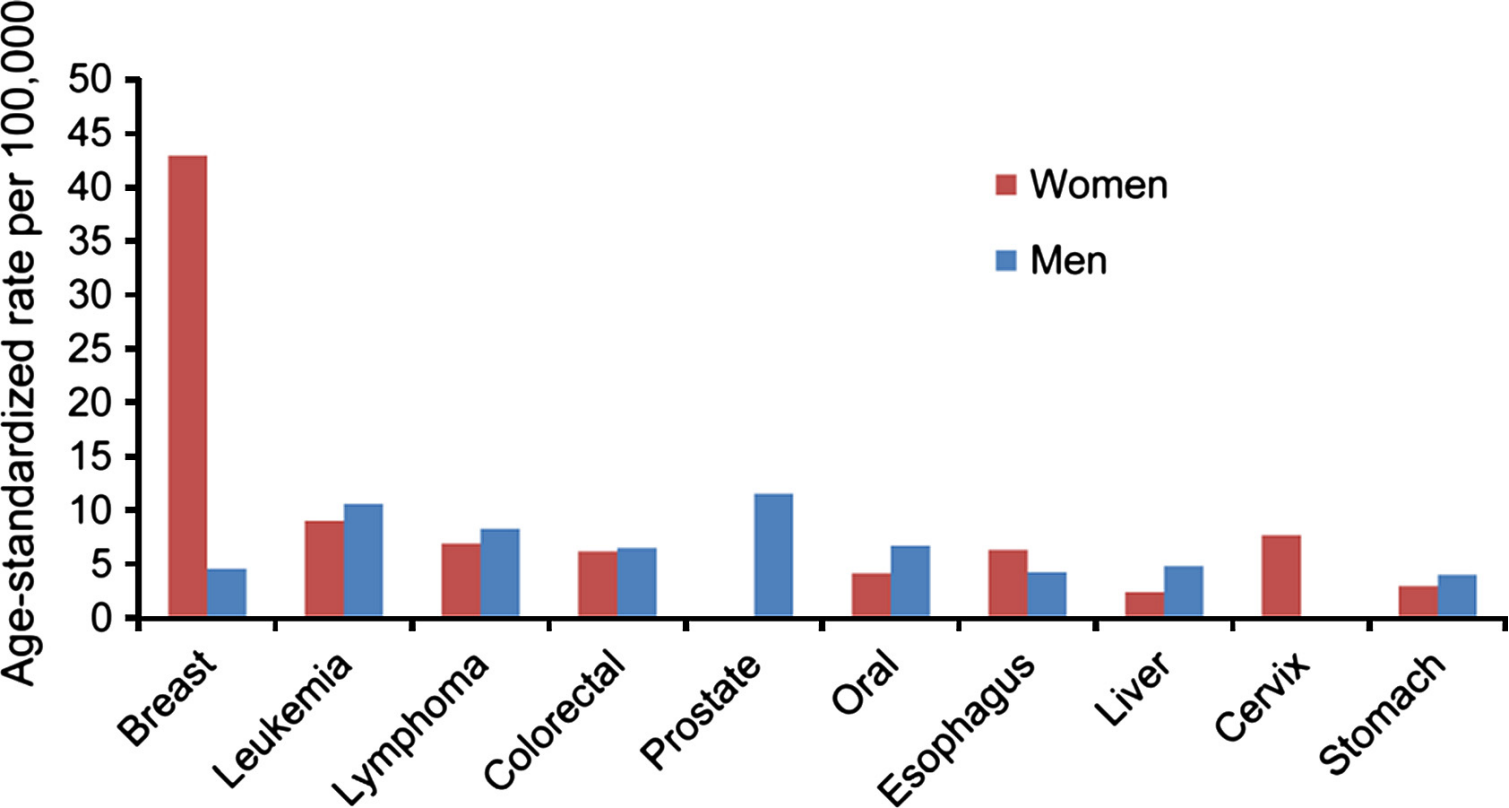
Age-standardized rate (ASR) of the most common cancer sites by gender, Khartoum, Sudan: 2009–2010. *N* = 6710.

During the study period in Khartoum, women had a higher ASR (124.3 per 100,000 population), using Sudan 2010 estimated population (both sexes combined) as the standard population, than men (ASR = 90.8 per 100,000) for all cancer sites combined (Table3). The estimated number of breast cancer cases was much higher than the other most common cancers (Table3). Figure6 compares the ASR (standard population: 2010 Sudan population) of the most common cancer sties between women and men. The ASRs, using the 1966 and 2000 WSPs, for the most common cancer sites by gender are present in Table4. The differences in the ASRs derived from the three standard populations indicate that the age distributions are different among these populations. Our results suggest that the 2000 World Standard Population is slightly older than the 1966 World Standard Population, while the 2010 Sudan Population is much younger than the two.

**Table 3:** Age-standardized rate (ASR) and estimated number of incident cancer cases for the most common cancer sites by gender in Khartoum, Sudan: 2009–2010.

Primary cancer site	ASR^1^			Estimated number of incident cancer cases^2^		
	Women	Men	Total	Women	Men	Total
Breast	41.1	4.4	21.1	1112	133	1215
Leukemia	8.6	10.2	9.4	234	310	543
Lymphoma	6.6	7.9	7.3	179	242	422
Prostate	–	6.0	–	–	185	–
Colorectal	5.9	6.2	6.0	159	189	348
Esophagus	6.0	4.1	5.0	163	124	286
Oral	4.0	6.4	5.3	107	196	305
Ovary	7.0	–	–	188	–	–
Liver	2.3	4.6	3.6	62	141	205
Cervix	7.4	–	–	199	–	–
Stomach	2.8	3.8	3.4	76	117	194
Lung	1.8	3.6	2.8	48	109	159
Total^3^	124.3	90.8	106.0	3361	2772	6105

Sudan 2010 estimated population, both sexes combined, was used as the standard population in computing ASR. *N* = 6710.

1 Per 100,000 population.

2 Estimated using ASR and Khartoum 2009 population.

3 All cancer sites combined.

**Table 4:** Age-standardized rates (per 100,000) for the most common cancer sites by gender in Khartoum, Sudan: 2009–2010, using the 1966 and 2000 World Standard Population (WSP). *N* = 6710.

Primary cancer site	1966 WSP			2000 WSP		
	Women	Men	Total	Women	Men	Total
Breast	60.8	6.5	31.1	66.8	7.1	34.1
Leukemia	10.7	12.5	11.6	11.2	12.9	12.1
Lymphoma	9.4	11.5	10.6	10.1	12.4	11.3
Prostate	–	22.1	–	–	25.2	–
Colorectal	9.6	9.7	9.6	10.7	10.8	10.7
Esophagus	10.2	7.5	8.7	11.3	8.5	9.7
Oral	6.1	10.7	8.7	6.8	11.8	9.5
Ovary	10.8	–	–	11.8	–	–
Liver	3.9	8.0	6.1	4.3	8.9	6.8
Cervix	12.2	–	–	13.4	–	–
Stomach	4.9	6.4	5.7	5.4	7.1	6.3
Lung	3.3	6.7	5.1	3.7	7.5	5.8
Total^1^	188.6	145.4	165.0	206.3	160.0	181.0

1 All cancer sites combined.

## Discussion

This study focused on the cancer cases diagnosed and treated in Khartoum State during the period 2009–2010. It is the first study to report on incident cancer cases abstracted and recorded by the first Sudan National Cancer Registry. During the study period, 6771 cases were recorded for Khartoum State. Although, the NCR provided the best available population-based estimations to the cancer profile in Khartoum State, different sources of bias may have occurred. First, stigmatization and poverty contribute to patients seeking other alternative treatments before pursuing treatment at proper cancer treatment institutions; leading to some cases to remain undiagnosed or untreated. Second, most of cancer-treating facilities, particularly radiotherapy facility, are at Khartoum city. Hence, some of these registered cases may have come from out of the state. Most out-of-state patients may use in-state relative's address or phone number in Khartoum for convenience. This may have led to overestimation of the cancer incidences. Third, most patients do not have birth certificates and therefore their age were approximations. This is especially true for elderly patients. Further, there was a possibility of under registration of clinically diagnosed cases in older individuals as well. Elderly patients were often not fully examined and were only treated symptomatically at the primary care level as outpatients or die before reaching a cancer specialized institution. Therefore, their medical records, if available, may not be complete or accurate. These limitations, such as limited accessibility to health care and inaccuracy of residency and age were reported as well from cancer registries of other African countries 5–8,11.

There are several means to measure the quality and completeness of data from a cancer registry 9. These include the percentages of registered cancer cases by death certificate only, and morphological verification, and percent missing. A DCO% of 0%, a MV% of 100%, and a percent missing of 0% denote a perfect data quality and completeness. However, acceptable limits for data quality indicators based on international standard by IARC in Cancer Incidence in Five Continents Volume IX (CI5-IX), are MV% between 75% and 98%, DCO% <10%, and percent missing of less than 10% 10. Although, a near-perfect value for MV% is desirable in developed countries, perfect values in developing countries means overreliance on pathological services and lack of examining other diagnostics means. Our data showed that 60% of the cases in NCR were morphologically verified. This value is less than internationally recommended, that is, the registry did not meet international data quality requirements. Many reasons could be cited and may include the young age of the registry, leaning curve required by the staff for data entry, and processing or different diagnostic sources were used for case accrual. However, the overall data quality of the NCR, 60% MV, 0.04% DCO, and 12% missing, was comparable to data quality of well-established cancer registries in East African countries such as Uganda (59% MV) 11 and Zimbabwe (64% MV) 12. However, it is lower than that reported by West African countries. For Abuja and Ibadan, Nigeria, the MV% were 94% and 84%, respectively, and for Abidjan, Ivory Coast, the MV% was 81.82% 5,8. Similarly, it was lower compared to North Africa countries' cancer registries. The MV% was 79% in Benghazi, Libya, 2003; 88% in Algeria, Algeria, 1993–1997; 90% in Tunis, Tunisia, 1994; and 82% in Garbiah, Egypt, 1999–2001 13–15.

During the study period, more women have cancer than men. The women to men ratio were 1.2:1.0. However, the increase is very modest compared to what was reported by Jedy-Agba et al. for Nigerian as the number of women was twice the number of men 5. The increase in the number of women cancer incidence may be attributed to the number of breast cancer registered and under diagnosis of prostate cancer.

Breast cancer is the most common cancer in Sudanese women living in Khartoum State. The incidence rate of breast cancer was substantially higher than other types of cancer in adults aged 25–64 years. The ASRs of breast cancer in women living in Khartoum State, using the 1966 and 2000 WSP, were 60.8 and 66.8 per 100,000, respectively, which were higher than what reported in black women in Harare, Zimbabwe (46.8 per 100,000, 2006–2010), and in Kampala, Uganda (31.0 per 100,000, 1991–2006) in East Africa 12,16. The incidence rate of breast cancer in women in Khartoum was also higher compared to North Africa, such as in Benghazi, Libya with an ASR of 22.9 per 100,000 in 2003, 24.1 per 100,000 in Tunis, Tunisia (1993–1997), and 49.6 per 100,000 in Garbiah, Egypt (1999–2000) 13–15. However, our findings were similar to those reported in Nigerian women living in Abuja (64.6 per 100,000, 2009–2010) or living in Ibadan (52.0 per 100,000, 2009–2010) 5. Additionally, the ASR of breast cancer estimated in our study was higher than that estimated by Globocan 2008 for the entire Sudan (24.0 per 100,000), as well as Eastern (19.3 per 100,000) and Northern (32.7 per 100,000) Africa 17. Previous studies have found breast cancer incidence is lower in East African women than that in West African women 18, therefore the relatively high incidence rate of breast cancer in Khartoum is quite surprising. Many risk factors associated with urbanization and economic development were cited to contribute to increase in breast cancer and these include early menarche, late childbearing, having fewer children, obesity, and increased awareness and detection 12. Khartoum population, on average, has high percentage of educated women. Increase in infertility and overweight which till recently is a sign of beauty and wealth in these communities may contribute to the increase in the breast cancer incidence in Khartoum.

Prostate cancer in sub-Saharan Africa emerged as the most common cancer and registries record suggests that the disease is increasing in prevalence. Prostate cancer ranked fourth among all cancer sites in Khartoum. However, by gender it ranked first among Sudanese men. It had the highest age-specific rate in seniors aged 65 years and older. The ASRs of prostate cancer among men in Khartoum State were 22.1 (1966 WSP) and 25.2 per 100,000 (2000 WSP), which were relatively lower compared to other African countries. Recent data from Harare, Zimbabwe (1991–2010); Kampala, Uganda (1991–2006); Ibidjan, Ivory Coast (1995–1997); and Ibadan and Abuja, Nigeria reported much higher ASRs for prostate cancer 73.0, 39.6, 31.4, 17.4, and 25.9 per 100,000 (1966 WSP), respectively 5,8,12,16. Therefore, most African populations reported higher rates of prostate cancer compared to Khartoum State. However, our data agrees with the low incidence rates reported in Red Sea and Western states of the Sudan 19,20. The low incidence rate may reflect less diagnosis and lack of screening programs than disease occurrence. Risk factors for prostate cancer in Sudan was examined by Hamad and Abuidris in Gezira state and found to include age, education level, occupation, unhealthy habit such as smoking and high fat intake and obesity, and were found to be similar to other parts of Africa 21.

Although cervical cancer used to be the most common cancer among women in sub-Saharan African countries, currently the picture is changing and surpassed by breast cancer. Similarly, in Khartoum State cervical cancer was the second most diagnosed cancer in women after breast cancer. The highest incidence rate of cervical cancer was observed in women aged between 55 and 64 years followed by women aged 65 years and older. The ASRs were 12.2 per 100,000 (1966 WSP) and 13.4 per 100,000 (2000 WSP) and were higher than the estimate for Sudan (ASR 7.0 per 100,000) for the period 1998–2002 17. However, compared to the neighboring countries in East African, the incidence rate of cervical cancer in Khartoum was low. The ASR of cervical cancer in Harare, Zimbabwe (2006–2010) was 103.8 per 100,000 12 and was 52.4 per 100,000 in Kampala, Uganda 16. Furthermore, it was lower than what were reported by West African countries. For example, the ASR of cervical cancer reported in Ibidjan, Ivory Coast (1995–1997) was 6.8 per 100,000, was 36.0 per 100,000 in Ibadan, Nigeria, and was 30.3 per 100,000 in Abuja, Nigeria 5,8. The ASR reported for this study is comparable to what was reported for Black American women in the United States (ASR 12.0 per 100,000, 2009) (www.cdc.gov/cervical/statisitcs.html.). The age of patients presenting with cervical cancer is >55 years. This may have contributed to under reporting as Sudanese women in that age group is usually not open about their gynecological problems with family (husband or sons are the ones financially responsible party for treatment). Furthermore, other factors such as lack of screening tests and awareness, and low prevalence of Human Papilloma Virus (HPV) and human immunodeficiency virus (HIV) infection in general population may contribute to this low level of cervical cancer cases. While HPV and HIV are known risk factors for cervical cancer, few studies examined the association between HPV infection and cervical cancer among Sudanese women 22,23. Similarly, there is a knowledge gap about the association between HIV infection and cervical cancer. According to the estimate conducted in 2011, the adult HIV prevalence of Sudan was 0.53%, which was much lower than the prevalence of 8.4% reported among Ugandan women. There are about 12 million individuals living with HIV/AIDs in Uganda, while about 260 thousands in Sudan in 2009 (http://ciaworldfactbook.us/).

Cancer in children less than 15 years old constituted about 7% of the cancer cases recorded by the NCR. Similar finding was reported in Malawi 24. In Khartoum State, the most common cancer in children were leukemia, lymphoma, and cancer of the eye, bone, kidney, brain, breast, oral, liver, and stomach. Lymphomas were mostly non-Hodgkin's lymphoma and eye tumors were mostly retinoblastoma. Some of these findings are similar to the findings from a previous study in Sudan where lymphoma, leukemia, and Wilms' tumors were reported to be the three most common cancers in this age group 25 and similar pattern has been seen in North African countries such as Tunisia 26 and Morocco 27. The Khartoum childhood cancer profile is different from that in neighboring sub-Saharan African countries, which were mostly dominated by Burkett lymphoma and Kaposi sarcoma 28.

## Conclusion

This is the first report in cancer incidence by the newly established national cancer registry in Sudan. Despite the study limitations, the NCR data gave a fair representation of cancer profile of Khartoum State and underscored the need for high-quality cancer registries in Sudan. The cancer incidence data indicated that prostate and breast cancers as the most commonly diagnosed disease in men and women in Khartoum while cancer of the cervix trailed behind portraying a cancer picture similar to that of the developed world.

## Conflict of Interest

None declared.
